# 多发性骨髓瘤克隆演变研究进展

**DOI:** 10.3760/cma.j.issn.0253-2727.2021.07.017

**Published:** 2021-07

**Authors:** 轶 王, 刚 安, 录贵 邱

**Affiliations:** 中国医学科学院、北京协和医学院血液病医院（中国医学科学院血液学研究所），实验血液学国家重点实验室，国家血液系统疾病临床医学研究中心，天津 300020 State Key Laboratory of Experimental Hematology, National Clinical Research Center for Blood Diseases, Institute of Hematology & Blood Diseases Hospital, Chinese Academy of Medical Sciences & Peking Union Medical College, Tianjin 300020, China

多发性骨髓瘤（MM）是恶性浆细胞克隆性增殖所致疾病[1]，由意义未明的单克隆免疫球蛋白血症（MGUS）、冒烟型骨髓瘤（SMM）逐步进展而来，是研究肿瘤克隆演变的重要模型。越来越多的研究揭示MM具备基因组高度不稳定的内部特征和对微环境高度依赖的外部特性[1]–[3]，两者共同参与了MM的发生及发展。本文通过归纳总结近年来相关文献，分析MM克隆异质性、克隆演变的发生机制及模式，以期更好地认识MM的生物学特点，为诊疗及预后评估提供新思路。

一、MM的克隆异质性

1976年，Nowell等[4]最先提出“肿瘤自然选择论”，认为肿瘤存在克隆异质性，在整个病程中不断发生克隆演变。存在克隆异质性是MM的重要特征，也是其发生克隆演变的基础。

MM克隆异质性的内涵包括以下几点：①不同患者之间：每例MM患者中位突变率为1.6个突变/1 000 000碱基，遗传学差异明显[2]；②同一患者不同时期：随着疾病进展，遗传学异常（CA）增多，克隆突变谱发生改变[5]；③同一患者不同部位：全外显子测序（WES）研究显示不同取样部位的CA构成有明显差异[6]，其他研究也证实髓内病变及髓外病变的生物学特性和对治疗的反应不同[7]；④同一患者同一时期：Lohr等[8]的研究表明，平均每例MM患者可检测到5个不同克隆，部分患者可多达10个。

常见肿瘤的异质性由低至高排序为：一般的血液肿瘤（如急性髓系白血病、毛细胞白血病等）、MM、上皮来源恶性肿瘤（如肺癌、黑色素瘤等）[9]。

二、MM克隆演变的发生机制

1. 基因组不稳定性：基因组不稳定性是MM克隆异质性的主要原因，具备基因组不稳定性的细胞在分裂、分化过程中更易产生新的遗传学改变并发生演变[10]。MM的基因组不稳定性主要体现在染色体异常和高频突变上，其中染色体异常又分为数量异常及结构异常[3]。相同或不同类别的不稳定因素可相互诱发和积累，加剧基因组的不稳定性。

（1）染色体数量异常：绝大多数MM患者都存在染色体拷贝数变异（CNVs）[2]，相关机制暂不明确。近期对SMM和MM全基因组测序（WGS）或WES的研究结果显示CNVs出现的时机不同，支持其在病程中持续积累[5],[11]。

涉及整个染色体的CNVs主要包括超二倍体（HRD，以奇数染色体三体为主要特征，染色体数48～74）和亚二倍体（染色体数<45）。它们通常出现在疾病早期，HRD与预后较好相关，而亚二倍体患者预后较差[2]。此外，四倍体也可见于约3％的SMM患者和10％的MM患者[5],[11]。

MM的CNVs还可体现在染色体臂或更小的结构水平上。拓扑关联域（TAD）在生理条件下是基因组元素相互作用的染色体结构域，促进转录调控。Wu等[12]的研究表明，MM中30％的CNVs发生在TAD边界或附近，限制基因表达；其余70％则发生在TAD内，可发挥新的调控作用。常见并能引发更多基因组不稳定性的CNVs有：①1q扩增，发生在35％～50％的MM患者中[2],[13]，多由中心体周围染色体不稳定所致，常见的形式包括1q21扩增、1q的“跳跃易位”等。其中1q21扩增与不良预后相关，可通过上调CKS1B表达引起更多遗传学改变[14]。②1p缺失，发生率约为30％，可导致细胞周期蛋白依赖激酶抑制基因CDKN2C缺失，使过多细胞由G_1_期进入S期[2]；也可使MTF2表达下调抑制p53的作用，导致细胞凋亡受抑和耐药[15]。1p缺失是SMM进展至MM的危险因素，对MM患者的生存有负面影响[11],[16]。③13q缺失或13号染色体单体，可见于45％～50％的MM患者，导致含RB1在内的多个肿瘤抑制基因缺失并提高基因组的不稳定性，主要作用机制为细胞周期失调控。目前认为13q缺失无独立预后意义，但与MGUS进展至MM相关，且常与17p缺失、t（4;14）等高危CA共存[2]–[3],[17]。④17p缺失，大约10％初诊MM患者可出现17p缺失，当疾病进展至终末阶段，其出现率可达80％。主要涉及的基因为TP53[18]，影响DNA修复、细胞周期暂停和凋亡。25％～40％的17p缺失患者同时合并TP53突变，此类患者更高危[17],[19]。

（2）染色体结构改变：染色体结构改变表现为平衡/不平衡易位、串联重复、倒位或缺失等，MM以染色体易位为主。B细胞需要在生发中心经历免疫球蛋白基因重排、体细胞高频突变及类别转换才能产生多种高特异性抗体，上述各过程均需要活化诱导的胞苷脱氨酶（AID）参与介导DNA双链解聚、重组，这是MM发生染色体结构改变和部分基因突变的主要原因[3]。

涉及免疫球蛋白重链（IGH）基因的染色体易位约占MM所有易位的90％，常见的易位形式、发生率和伴侣基因分别为：t（4;14）（约占15％），涉及MMSET/FGFR3；t（6;14）（占1％～2％），涉及CCND3；t（11;14）（占15％～20％），涉及CCND1；t（14;16）（约占5％），涉及MAF；t（14;20）（约占1％），涉及MAFB[2],[20]。这些易位使得伴侣分子处于IGH增强子控制之下，高表达特定癌基因。作为MM的两种初级CA，IGH易位与HRD极少共存[5]，但两者均可作用于细胞周期蛋白通路，通过增加CyclinD表达促进肿瘤细胞增殖并提高基因组的不稳定性[3]。除了IGH易位，MYC易位或局部扩增也是重要的染色体结构异常，能通过多种效应影响细胞周期并导致染色体异常[21]–[22]。

染色体碎裂和染色体丛通过非同源染色体断端联合产生广泛重排，也是导致基因组不稳定及肿瘤发生、发展的关键结构变异[23]–[24]。染色体碎裂最多累及2条染色体，是由于微核核膜异常导致被包裹的染色体在有丝分裂时暴露于胞质并发生碎裂[25]；染色体丛则涉及3条及以上染色体，发生机制有待更多研究阐明。近期研究显示，染色体碎裂和染色体丛在初诊MM中的发生率分别为21％和10％～46％，与疾病进展、APOBEC高频突变相关[5],[26]

（3）基因突变：与毛细胞白血病（BRAF V600E）、慢性粒细胞白血病（BCR-ABL）不同，MM尚未发现驱动性基因突变。随着大规模二代测序的应用，常见突变及其信号通路被逐步证实[8],[27]–[28]。MAPK通路是最常累及的信号通路（约40％），KRAS、NRAS和BRAF等均可发生突变，与疾病进展相关。突变也常发生于NF-κB信号通路（约20％），突变基因可为TRAF3、CYLD、LTB、IKBKB、BIRC2等。TP53、ATM、ATR等DNA修复信号通路基因突变率约15％。B细胞分化通路基因突变，如PRDM1、IRF4、LTB和SP140等突变在部分研究中被认为是重视性基因。其他突变如DIS3、FAM46C等重现率较高，分别参与RNA的合成、清除及蛋白质翻译，一般被认为是抑癌基因。

这些突变不断产生和积累，特定的突变模式是基因组不稳定性标志。AID参与的DNA双链的解聚、重组是产生基因突变的重要机制。生理情况下，kataegis是一种易发生在IGH或免疫球蛋白轻链（IGK、IGL）位点周围的局部超突变域，但超过60％的MM患者除外这些区域仍有不少于1个kataegis结构，进一步分析显示AID是该结构区域产生基因突变的主要原因且与非IGH/IGL/IGK重排、APOBEC突变密切相关[29]。APOBEC是一种胞苷脱氨酶，功能异常时可导致DNA高频突变，与高突变负荷及总突变数呈正相关。在SMM和MM中，APOBEC突变分别与疾病进展至有症状阶段和不良预后相关，常出现在合并t（14;16）、t（14;20）的患者中[11],[30]。目前的研究结果认为，AID在疾病早期对突变谱的塑造有重要作用，而APOBEC则驱动后续基因突变和病情进展。

2. 微环境作用：微环境的改变可早于CA，形成“癌前微环境状态”，促进细胞的恶性转变及增殖，决定肿瘤细胞表型和局部优势克隆[31]。肿瘤细胞也可反向作用于微环境，使其更利于自身存活及进展。微环境是MM发生克隆演变的重要外部因素。

肿瘤进展有赖于免疫逃逸，骨髓微环境异常可削弱免疫系统抗肿瘤效应，有助于恶性细胞存活和增殖。从MGUS到MM，免疫微环境中抗原呈递减少，免疫效应细胞失能，免疫抑制细胞增多[32]。有研究发现在MM微环境中TH17增多并抑制免疫监视[33]，MM相关CD8^+^T细胞则高表达PD-1导致免疫耐受[34]。参与MM骨病的破骨细胞也可通过高表达β-半乳糖苷结合蛋白Galectin-9、CD38、PD-L1或分泌增殖诱导配体APRIL等促进T细胞凋亡及抑制T细胞介导的细胞毒性[35]。

MM患者骨髓的氧含量较正常对照更低，提示低氧也在疾病发生发展中起到重要作用。低氧通过增加DNA损伤并抑制错误修复造成基因组不稳定，也可因氧化代谢改变影响表观遗传学对基因的调控和肿瘤细胞对化疗的敏感性[36]。此外，蛋白组学研究表明非MM、MGUS和MM患者骨髓标本细胞外基质构成不同，其也参与MM克隆演变[37]。

3. 治疗选择压力：近年来，以蛋白酶体抑制剂（PIs）、免疫抑制剂（IMiDs）为代表的新药有效延长了MM患者的生存[38]–[39]。但治疗作为一种外在压力，在产生疗效的同时也重塑了MM细胞及其微环境，诱发克隆演变，导致疾病进展或复发。

部分研究认为，治疗可以诱发新的表型改变，使疾病向耐药演化。Barrio等[40]的研究表明，在长期暴露于硼替佐米的细胞系中可检测到亲代细胞中没有的突变，其通过阻止未折叠蛋白积聚和降低内质网应激信号使MM细胞对PIs耐药。而cereblon蛋白低表达、CD44高表达及上调MAPK/ERK信号通路活性与IMiDs耐药有关[41]–[42]。

更多研究则支持治疗对克隆演变的推动主要依靠对已有克隆的筛选。数个配对标本研究证实[19],[43]–[44]，治疗后复发的肿瘤细胞克隆构成发生改变，高侵袭性或耐药的克隆比例明显增高甚至成为优势克隆。对上述文献总结发现，易于经治疗筛选的细胞多伴有以下特征：①与细胞周期、DNA复制相关的基因表达上调；②合并继发负性CNVs，如17p缺失、1q扩增等；③PI3K/RAS、NOTCH、DNA损伤修复等信号通路激活；④TP53、RB1等抑癌基因双等位基因失活事件增多；⑤出现新的突变模式。不同研究对比显示，MM克隆变化程度与治疗强度呈正相关，且需改变克隆结构才能导致演变。

事实上，治疗所致克隆演变是一种持续渐进的过程，治疗后的微小残留病（MRD）水平即可显示克隆变化，监测MRD的数量和质量均非常重要。Paíno等[45]和Paiva等[46]的研究表明，治疗后MRD阳性的患者可以检测到流式表型及遗传学克隆漂移。近期，An等[39]发现44.4％的MRD阴性患者的浆细胞仍可通过FISH检测到CA，且初诊时合并高危或多个CA的患者易发生治疗相关克隆演变，生存更差。

三、MM克隆演变模式

近年来，“肿瘤自然选择论”在多种肿瘤中被证实，克隆演变赋予了疾病新的特征，而总结演变模式有助于理解疾病发展的本质和规律。

目前主要通过遗传学检查监测克隆演变，可选用的手段包括WES、WGS、靶向测序和FISH等，少数研究也采用流式细胞术等其他技术。克隆构成改变是判断发生克隆演变的重要依据。多项研究归纳了MM的克隆演变模式[19],[28],[44],[47]，主要分为以下4种：①稳定型，克隆构成基本不变；②分化型，优势克隆变化，但无新克隆出现；③线型，不断出现新的克隆并成为优势克隆，既往克隆仍然存在；④分枝型，新克隆成为优势克隆，既往克隆消失。

MM演变模式通常与特定的疾病危险分层或CA相关[27],[39],[46]：低危患者多以稳定型或分化型模式发生克隆演变；线型或分枝型演变模式则常见于高危患者，尤其是以髓外病变为主要表现的复发患者；t（11;14）常见于稳定型模式，TP53、FAM46C功能缺失则多见于分枝型模式。此外，既往治疗取得更好疗效的患者在进展时多发生分枝型演变[47]。

四、MM克隆演变的意义

异质性及克隆演变是恶性疾病复发难治的根本原因，加深对其理解有助于明确MM的诊疗是多层次的动态过程。目前基于生化指标、CA及蛋白表达等的危险分层体系已能够较好预测患者预后[48]，但空间异质性的存在提示单一部位取样可能会误判病情或不能匹配准确的分层治疗策略[6]。并且，MM复发或经过治疗后大多发生了克隆演变[19],[43]，此时初诊的疾病状态并不能反映即时病情或预测未来发展趋势，应重新进行遗传学检查或预后分层评估。通过不同克隆演变模式复发的患者宜采取的治疗策略也不同：既往治疗对稳定型演变患者可能依然有效；发生分枝型或线型演变的患者则需选用不同作用机制的药物，甚至采取全新治疗策略以杀灭耐药或新发克隆。

出现症状是MM启动治疗的“标准”时期，而克隆演变的存在提示可能存在更佳治疗时机。MM主要以分枝型模式发生演变[19]，逐渐具备更复杂的克隆结构，容易因无法杀灭所有肿瘤克隆导致反复进展。Dutta等[49]的研究认为肿瘤克隆在从MGUS或SMM进展至MM的过程中基本保持稳定，因此SMM可能是最佳治疗时机。目前SMM治疗思路主要分为两类，一类是以免疫治疗为基础的治疗策略，以延缓疾病进展为主要目的，但可能导致残存肿瘤细胞最终发展为难治性克隆；另一类则是早期予包括移植在内的系统性治疗，尽管不良反应大但反应率高，有潜在治愈可能。MM治疗时机是否需要提前、如果提前应采用何种治疗策略有待后续更多研究得出结论。

MM的异质性及克隆演变也是精准治疗的难点，多药联合和免疫治疗是重要的突破口。免疫治疗利用机体免疫系统攻击肿瘤，是近年来肿瘤治疗领域的重大进展，其多重作用机制、非单一作用位点的特征满足MM治疗策略的需求。Alcyone、CASSIOPEIA等研究提示以CD38单抗为基础的联合治疗或将成为一线治疗方案[50]–[51]，而CAR-T细胞疗法和靶向BCMA的抗体偶联药物也在多个复发难治MM的Ⅰ期/Ⅱ期临床试验或临床前研究中显示出了良好的安全性和有效性[52]–[53]。免疫治疗的蓬勃发展将为MM带来更多更有效的治疗选择。

五、小结与展望

MM是一组高度异质性疾病，越来越多的研究证实克隆演变在其发生发展中发挥着重要作用。探究MM克隆演变的发生机制及具体模式有助于我们理解MM的生物学特性，并从危险分层、治疗时机和策略等维度优化诊疗策略。
